# Ultrasound-guided continuous radiofrequency ablation of the proximal greater occipital nerve is effective in refractory occipital neuralgia: a retrospective cohort study

**DOI:** 10.1055/s-0045-1806813

**Published:** 2025-04-02

**Authors:** Suna Aşkın Turan, Şenay Aydın, Ezgi Can

**Affiliations:** 1 University of Health Sciences, Mersin City Training and Research Hospital, Department of Pain Medicine, Mersin, Republic of Türkiye.; 2 University of Health Sciences, Yedikule Chest Disease and Surgery Training and Research Hospital, Department of Neurology, Istanbul, Republic of Türkiye.; 3 University of Health Sciences, Ankara Etlik City Training and Research Hospital, Department of Pain Medicine, Ankara, Republic of Türkiye.

**Keywords:** Neuralgia, Headache, Radiofrequency Ablation, Nerve Block, Ultrasonography, Interventional, Pain Measurement, Treatment Outcome

## Abstract

**Background:**

Pain in occipital neuralgia (ON) originates from the upper cervical nerves converging with the trigeminal complex. Greater occipital nerve (GON) blocks and radiofrequency treatments can be used in refractory ON.

**Objective:**

To assess the efficacy of ultrasound-guided proximal greater occipital nerve (PGON) continous radiofrequency ablation (CRFA) in ON throughout 1 year.

**Methods:**

We analyzed data from medical records and headache diaries. Before the intervention and 1, 3, 6, and 12 months after the intervention, at each appointment we evaluated the headache intensity through the 11-point Numeric Rating Scale (NRS-11), the headache disability, through the Six-Item Headache Impact Test (HIT-6), as well as the headache days per month. Treatment efficacy was determined by NRS-11 score < 4 at 12 months.

**Results:**

A total of 18 patients were analyzed. The mean initial NRS-11 score was of 8.78 ± 0.732. At least 50% of pain reduction was noted in all patients at 6 months, and in 66% patients at 12 months. The frequency of attacks was correlated to poor response (r = 0.598; *p* = 0.009). The efficacy of the diagnostic block was correlated to successful response (r = -0.789; *p* = 0.001). For the categorical variables, the electric shock pain was associated with NRS-11 score ≥ 4 ( *p* = 0.041), and lancinating pain was associated with NRS-11 score < 4 at 12 months ( *p* = 0.031).

**Conclusion:**

Ultrasound-guided PGON CRFA in refractory ON significantly reduced pain for up to 1 year. The initial frequency of attacks, electric shock like pain, and reduced response to diagnostic block were associated with reduced response.

## INTRODUCTION

Occipital neuralgia (ON) is a unilateral or bilateral paroxysmal shooting or stabbing pain that occurs in the posterior region of the scalp and is characterized by the distribution of the greater occipital nerve (GON), the lesser occipital nerve (LON), or the third occipital nerve (TON). 1 Tenderness over the affected nerve and occasionally decreased sensation or dysesthesia in the affected area are common symptoms of the disease. 2 It often causes severe pain that impairs function and quality of life. 3 Most cases of ON are idiopathic and have no clearly-defined anatomical cause. 2 Initially, conservative treatment approaches such as medication and physiotherapy are often used. If conservative measures fail to alleviate ON, interventional treatments are available. 3 4

Radiofrequency ablation (RFA) has been shown to be effective in ON in previous studies. 4 Three RFA techniques have been used to treat ON, including continuous radiofrequency ablation (CRFA), pulsed radiofrequency (PRF) and cooled RFA. 4 However, the mechanism of action of the three RFA techniques is different, so their effectiveness in the treatment of ON may vary. In PRF, radio waves are delivered to the nerve in a pulsed sequence and at temperatures lower than 42°C. 4

Researchers have investigated the use of PRF for the treatment of ON. The GON PRF has led to a significant improvement in pain scores and quality of life, as well as a reduction in the use of pain medication. However, its effectiveness only lasted for a short period. 5 6 7 8 9 10 Continous RFA differs slightly from PRF in that it destroys tissue at a temperature higher than 42°C and interrupts pain by causing Wallerian degeneration. The pain interruption is longer and stronger than with PRF. However, side effects such as hyperesthesia, dysesthesia, and deafferentation pain have been observed more often than with PRF. 11 Cooled RFA is a relatively new technique developed to enlarge the lesion. 4

As the GON is a purely sensory nerve, CRFA could be an alternative treatment option for ON with longer duration and more efficient than PRF. 4 A few studies 11 12 13 14 have been published in the literature on the effectiveness of CRFA for ON under fluoroscopic control at the cervical 2 dorsal root ganglion (C2 DRG; defined as very proximal) or the external prominence (defined as distal), but these studies reported terrible side effects.

Ultrasound (US) could be used safely to determine the proximal GON (PGON) between the semispinalis capitis muscle and the obliquus inferior muscle at the level of C2. 15 Therefore, here, GON block might be more effective and safer. In the past, US-guided PGON block for chronic migraine, ON, and cervicogenic headache was considered more effective than distal GON (DGON) block performed at the external prominence. 16 17 18 In patients with chronic migraine, US-guided PRF of the PGON has already shown promise. 19 However, no study was found on the efficacy of US-guided CRFA of the PGON for ON.

Our hypothesis is that CRFA of the PGON might provide to ON patients a longer and more efficient treatment compared to previous studies, with fewer side effects. The main goal of the present study was to evaluate the efficacy of US-guided CRFA of the PGON in refractory ON throughout 1 year. The secondary aim was to determine the parameters related to treatment efficacy, which was defined by NRS score < 4 at 12 months follow-up. 20

## METHODS

### Standard protocol approval and patient consent

In accordance with the Declaration of Helsinki, the institutional Ethics Committee approved the current retrospective cohort study (reference number/date 2024-446/22.05.2024), with ClinicalTrials.gov Protocol Registration and Results System (PRS) NCT06458179. Informed consent was obtained from all participants.

### Study design and participants

#### *Inclusion criteria*

Between January 1, 2022, and April 30, 2023, all patients with primary unilateral ON who underwent US-guided diagnostic occipital nerve block and subsequent CRFA of the PGON in the Department of Pain Medicine and who were followed up for at least 1 year were identified and included.

#### *Exclusion citeria*

Patients who were unable to communicate, lacked documentation, or had inadequate follow-up were excluded. Patients were also excluded if they had received a new pain medication during the follow-up period that would have affected the assessment of their outcomes. Patients with bilateral ON, secondary ON (such as, cervical radiculopathy, infection, tumor, vascular compression of the nerve, musculoskeletal conditions such as C1-2 osteoarthritis etc.), other primary headaches, dermatitis or skin infections, and pacemakers were also excluded from the study.

#### *Diagnosis*

A diagnosis of ON was made based on the clinical history and neurological examination (including the Tinel test). A differential diagnosis of secondary ON was also made based on the results of hormonal and biochemical tests and imaging studies of the brain and cervical spine, such as X-rays or magnetic resonance imaging (MRI) scans, if needed. In total,3 patients had undergone brain and cervical spine MRI: 1 had cervical and 3-disc pathology, on MRI and were excluded from the study; the scans of the other 2 patients were within normal ranges.

**Figure 1:**
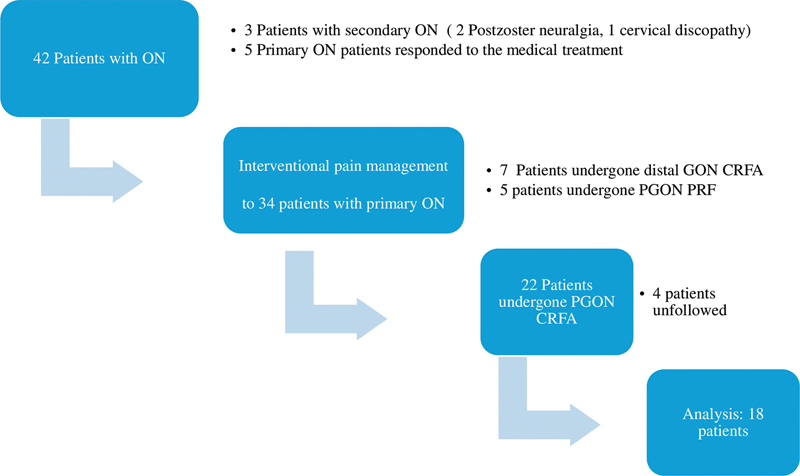
Abbreviations: ON, occipital neuralgia; GON, greater occipital nerve; CRFA, continuous radiofrequency ablation; PRF, pulsed radiofrequency. Flowchart of the study.

In total, 22 out of the 34 patients with primary ON received PGON CRFA therapy. We only analyzed 18 of these patients because 4 of them were excluded because they did did not return for follow-up: 2 patients did not come to the sixth monthly visit, 1 patient moved to a different city in the third month, and 1 patient did not come for a check-up after 12 months. None of these patients complained of any side effects during the follow-up. However, as they were unable to complete the 12-month follow-up, we excluded them from the analysis. Figure 1 shows the flow chart of the study.

### Medical treatment and intervention

Patients who did not respond to drug therapy received a diagnostic block (duloxetine 60 mg/day, pregabalin 300 mg/day, or carbamazepine 800 mg/day) and postural training against muscle tension for at least 3 months. If the response to the diagnostic block was higher than 50%, we initiated CRFA at the next visit.

In our routine outpatient pain medicine practice, we used a US machine with a 13 MHz to 5 MHz linear probe (Voluson E6, GE Healthcare, Chicago, IL, United States) to perform the diagnostic block and CRFA of the PGON without anesthesia in the operating room. During the diagnostic block, a 3-mL injection of bupivacaine 0.025% (Buvasin 0.5%, VEM İlaç, Istanbul, Republic of Türkiye) was administered. The block was considered successful if it could be maintained with more than 50% of relief for at least 1 hour. After 1 week of successful block, CRFA was performed.

**Figure 2:**
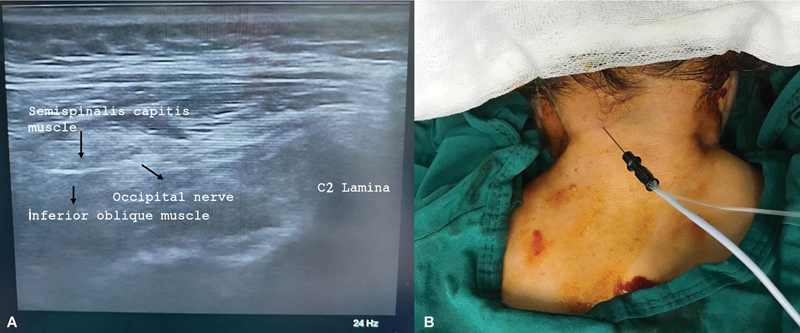
( **A** ) Sonoanatomy of the proximal greater occipital nerve (PGON); ( **B** ) placement of the radiofrequency needle.

Continous RFA was performed with a radiofrequency (RF) device (TOP Lesion Generator, model TLG-10, TOP Corporation, Tokyo, Japan) under US guidance in the operating room. The patient was monitored for vital signs in the prone position with the head slightly flexed. After disinfection, the probe was inserted transversely into the neck. After the occipital protuberance was visualized, the probe was pushed down to find the bifid spinous process of C2. The probe was lateralized (toward the affected side) to identify the semispinalis capitis muscle and the obliquus capitis inferior muscle ( Figure 2A ). In this position, the GON was placed between the muscles. A 21-G 5-mm RF needle with an active tip and a length of 60 mm (SC-K; TOP Neuropole, TOP Corporation) was used to treat the nerve from medial to lateral under ultrasound. ( Figure 2B ). Sensory stimulation was administered at 50 Hz for 1 ms to determine the location of the nerve after accurate placement of the needle tip. A voltage lower than 0.5 V was required to elicit paresthesia, pain or irritation in the nerve distribution during sensory stimulation. In addition, a motor stimulus was applied at 2 Hz for 1 ms and up to 2 V to check that no fasciculations were present. After negative aspiration of blood or fluid, 1 mL of 2% lidocaine was injected with the RF cannulas to relieve procedural pain. Continuous RFA was performed at a temperature of 60°C for 90 s. Immediately after the procedure, 2 mg of dexamethasone were injected into each lesion site to minimize the risk of neuritis. All adverse events after the procedure were documented for each subject.

### Outcome measures and follow-up

At the initial examination, the patient's demographic data and headache symptoms were thoroughly examined. In accordance with the recommendation of the Turkish National Headache and Pain Research Association, patients were asked to keep a headache diary for the duration of treatment and all subsequent appointments. Headache intensity, headache disability, and the number of headache days per month, as well as the use of non-steroidal anti-inflammatory drugs (NSAIDs), were determined from the diaries and discussed with the patient at each session.

The intensity of the headaches was measured at each appointment (before the intervention, and 1, 3, 6, and 12 months after the intervention) using the 11-point Numeric Rating Scale (NRS-11), a 1-dimensional scale that measures pain intensity in adults. The participant is asked to choose a number that best reflects the intensity of pain: 0 = no pain; 1 to 3 mild pain; 4 to 7 moderate pain; and 8 to 10 severe pain (with 10 indicating the worst pain imaginable). Treatment efficacy was defined as no pain or mild pain with NRS-11 score < 4 in the first year. 20

Headache disability was assessed at each appointment using the Six-Item Headache Impact Test (HIT-6). The final score on the HIT-6 ranges from 36 to 78 points: minimal influence (≤ 49 points); considerable impact (50–55 points); significant impact (56–59 points); and severe impact (≥ 60 points).The Turkish version of the scale was previously validated with a Cronbach's alpha of 0.87. 21

The primary and secondary endpoints were analyzed from the medical records and headache diaries with the data previously described.

### Statistical analysis

The statistical analyses were performed using the IBM SPSS Statistics for Windows (IBM Corp., Armonk, NY, United States), software, version 25.0. The percentages show the numerical values of the categorical variable, and the mean and standard deviation (SD) values for the continuous variables provide the statistical representation. For data with normal distribution, we used the Student's *t* -test to compare two groups, and analysis of variance (ANOVA) to compare three or more groups. For data that was not normally-distributed, the Mann-Whitney U-test was used. For the analysis of the categorical variables, the Chi-squared (χ ^2^ ) test or the Fisher's exact test were used. Correlations involving variables influencing the NRS-11 score at 12 months were analyzed using the Pearson correlation coefficient. Statistical significance was defined as *p* < 0.05.

## RESULTS

We analyzed 18 patients, 13 (72.2%) of whom were female subjects. Table 1 provides an overview of the patient characteristics.

**Table 1:** Sociodemographic and pain characteristics of the patients

Mean age in years (minimum–maximum)		48.39 ± 12.098 (28–72)
Gender: female – n (%)		13 (72.2%)
Mean Body Mass Index in kg/m ^2^ (minimum–maximum)		24.32 ± 3.042 (21.10–30.22)
Comorbidity: n (%)	Diabetes	4 (22.2)
	Hypertension	3 (16.7)
	Pulmonary disease	6 (33.3)
	Cardiac disease	4 (22.2)
	Thyroidal deficiency	5 (27.8)
	Depression	10 (55.6)
	Fibromyalgia	9 (50.0)
Side: n (%)	Right	10 (50.5)
	Left	8 (44.4)
Mean duration in years (minimum–maximum)		8.28 ± 3.707 (5–18)
Mean attack days/month (minimum–maximum)		12.11 ± 4.497 (5–20)
Mean number of attacks /day (minimum–maximum)		11.94 ± 7.033 (4–30)
Mean duration of the attacks in minutes (minimum–maximum)		68.89 ± 62.18 (5–180)
Mean pain intensity on the 11-point Numeric Rating Scale (minimum–maximum)		8.78 ± 0.732 (8–10)
Symptom: n (%)	Throbbing pain	12 (66.7)
	Electric shock-like pain	16 (88.9)
	Prickling pain	14 (77.8)
	Stabbing pain	15 (88.3)
	Lancinating pain	16 (88.9)
	Numbness	10 (55.6)
Signs: n (%)	Hyperesthesia	18 (100)
	Allodynia	9 (50.0)
	Tinel positivity	15 (83.3)
Rate of efficacy of the diagnostic block: n (%)	100%	2 (11.1)
	85–99%	8 (44.4)
	76–84%	3 (16.7)
	55–75%	5 (27.8)
Initial medical treatment: n (%)	Duloxetine	12 (66.7)
	Pregabalin	16 (88.9)
	Carbamazepine	18 (100)
Medical treatment at 12 months: n (%)	Duloxetine	4 (22.2)
	Pregabalin	1 (5.6)
	Carbamazepine	6: 33.3)
	(-)	7 (38.9)

Table 1 also provides the details of the ON findings. Overall, ON had a mean duration of 8.28 ± 3.707 years. Pain was consistently paroxysmal in all patients, with a mean duration of 68.89 ± 62.18 minutes per attack. The mean number of attacks was of 11.94 ± 7.033 per day, and the mean for the attack days per month was of 12.11 ± 4.497. The mean initial NRS-11 score ws of 8.78 ± 0.732. The diagnostic block yielded the following efficacy percentages: 100% efficacy −2 patients (11.1%); 85 to 99% efficacy – 8 patients (44.4%); 76 to 84% efficacy – 3 patients (16.7%); and 55 to 75% efficacy – 5 patients (27.8%). The prophylactic agents administered were as follows: carbamazepine 800 mg/day (18 patients; 100%), pregabalin 300 mg/day (16 subjects; 88.9%), and duloxetine 60 mg/day (12 patients; 66.7%).

The number of attacks, the use of NSAIDs for acute treatment per month, and the change within the scores on the NRS-11 and HIT-6 as a function of time are illustrated in Table 2 . The scores assessed during the appointments were lower than those at baseline. The relationship between time and the NRS-11 score was statistically significant ( *p* = 0.001). The HIT-6 score ( *p* = 0.001), the number of attacks ( *p* = 0.001), and the monthly intake of NSAIDs ( *p* = 0.001) also decreased after treatment at all appointments compared to baseline. At 12 months, 7 patients (38.9%) were taking no prophylaxis, 6 patients (33.3%), carbamazepine 400 mg/day, 4 patients (22.2%), duloxetine 30 mg/day, and 1 patient (5.6%), pregabalin 150 mg/day.

**Table 2:** Efficacy of RFA according to the NRS-11 score, monthly number of attacks, NSAIDs/month, and the HIT-6 score

	NRS-11	Attacks/month	NSAIDs/month	HIT-6
Baseline	8.777 ± 0.732 (8–10)	12.11 ± 4.497	13.222 ± 2.289	67.444 ± 2.175
1 month	1.111 ± 0.832 (0–3)	2.50 ± 1.654	0.000 ± 0.000	38.666 ± 2.169
3 months	1.666 ± 0.970 (0–3)	4.22 ± 1.801	1.556 ± 1.542	40.000 ± 2.376
6 months	2.500 ± 1.200 (0–4)	5.00 ± 1.029	1.388 ± 1.753	41.944 ± 2.460
12 months	3.722 ± 1.564 (1–6)	6.17 ± 0.707	4.666 ± 2.029	43.444 ± 2.974
*p*	0.001*	0.001*	0.001*	0.001*

Abbreviations: HIT-6, 6-Item Headache Impact Test; NRS-11, 11-Point Numeric Rating Scale; NSAIDs, nonsteroidal antiinflammatory drugs.

Notes: Anova test was used. Data are expressed as mean ± standard deviation (minimum–maximum) values; * statistically significant.

**Figure 3:**
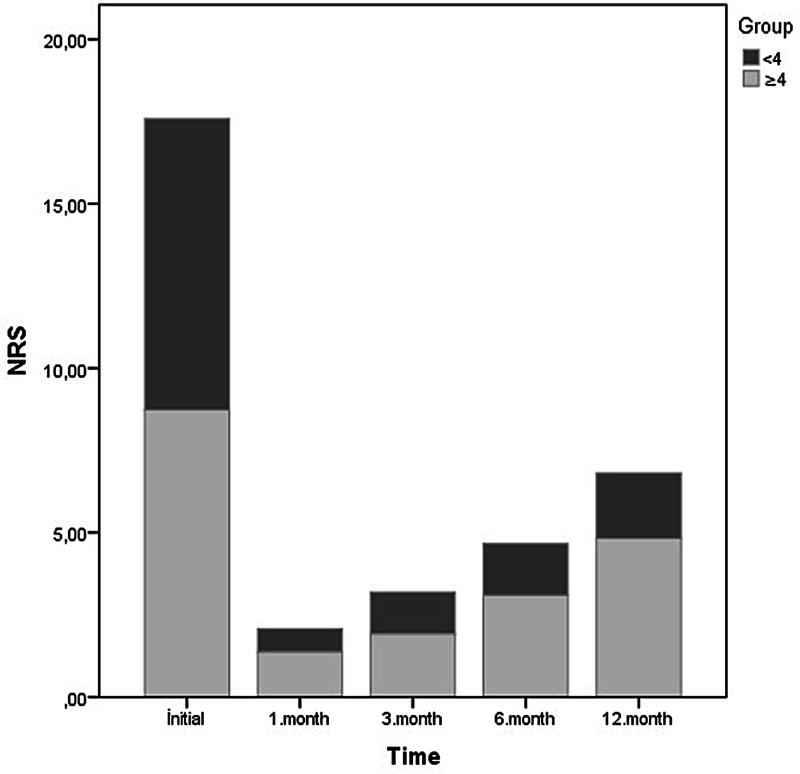
Mean scores on the 11-point Numeric Rating Scale (NRS-11) of the group with NRS-11 score < 4 and the group with NRS-11 score ≥ 4 at 12 months.

As previously mentioned, treatment efficacy was defined as NRS-11 score < 4 at 12 months. Table 3 shows treatment efficacy by month. The number of patients with NRS-11 score < 4 was of 18 (100%) at the first and third months, 14 (77.8%) at the sixth month, and 7 (38.9%) at 12 months. Figure 3 shows the mean NRS-11 scores for the group with NRS-11 score < 4 and the group with NRS-11 score ≥ 4 at 12 months. Pain reduction of at least 50% from baseline to the first, third, and sixth months was of 100%, and of 66% at month 12.

**Table 3:** Correlated factors affecting the NRS-11 score at 12 months

NRS-11 score at 12 months (score range: 1–6)	r	p
Age (years)	-0.239	0.339
BMI (kg/m ^2^ )	0.127	0.615
Pain duration (years)	0.176	0.484
Monthly number of attacks	0.598	0.009*
Attack duration (minutes)	0.163	0.518
Baseline NRS-11 score	-0.006	0.982
Diagnostic efficacy rate (%)	-0.784	0.001*

Abbreviations: BMI, body mass index; NRS-11, 11-Point Numeric Rating Scale.

Note: The Pearson's correlation coefficient was used in this analysis.

Table 3 shows the correlated factors influencing the NRS-11 score at month 12. With an increasing frequency of attacks per month, the correlation with the NRS-11 score also increased (r = 0.598; *p* = 0.009). The NRS-11 score decreased inversely as the percentage of effectiveness of the diagnostic block increased (r = -0.789; *p* = 0.001). For the categorical variables, electroshock pain was associated with an NRS-11 score ≥ 4 at 12 months ( *p* = 0.041). Lancinating pain was associated with an NRS-11 score < 4 at 12 months ( *p* = 0.031) ( Table 4 ).

**Table 4:** Comparison of categorical variables between the group with NRS-11 score < 4 and the group with NRS-11 score ≥ 4 at 12 months

Variable	Total	NRS-11 score < 4 (7 patients; 38.9%)	NRS-11 score ≥ 4 (11 patients; 61.1%)	*p*
Gender: female: n (%)	13 (72.2)	6 (85.7)	7 (63.6)	0.308
Depression: n (%)	10 (55.6)	5 (71.4)	5;45.5)	0.280
Fibromyalgia: n (%)	9 (50.0)	4 (57.1)	5 (45.5)	0.629
Positive Symptoms: n (%)	18 (100.0)	7;100.0)	11;100.0)	−
Throbbing pain	12 (66.7)	5 (71.4)	7 (63.6)	0.731
Electric shock-like pain	16 (88.9)	5 (71.4)	11;100.0)	0.041*
Prickling pain	14 (77.8)	7;100.0)	7 (63.6)	0.031*
Stabbing pain	15 (88.3)	6 (85.7)	9 (81.8)	0.827
Negative Symptoms: n (%)	16 (88.9)	7 (100.0)	9 (81.8)	0.145
Lancinating pain	16 (88.9)	7 (100.0)	9 (81.8)	0.145
Numbness	10 (55.6)	4 (57.1)	6 (54.5)	0.914

Abbreviation: NRS-11, 11-Point Numeric Rating Scale.

Notes: The Chi-squared test or the Fisher's exact test was used in this analysis; * statistically significant.

In total, 2 patients experienced complications or side effects. One of them reported that hypoesthesia persisted for 2 months, which was located in the posterior region of the scalp, where the treated GON was distributed. The hypoesthesia disappeared on its own in the third month. One patient reported dizziness and ataxia in the first week after CRFA. At that time, the neuroimaging and neurologic examinations were normal. The symptoms disappeared coincidentally in the second week. The rest of the patients did not experience any side effects after the procedure.

## DISCUSSION

As the authors hypothesized that US-guided CRFA of the PGON might be effective in the long term with fewer side effects, the findings of the current study support the hypothesis. The study highlights the benefits of CRFA targeting the PGON in ON patients, demonstrating both long-term efficacy and minimal, temporary side effects.

Pain reduction showed promising results: 100% of patients scored < 4 on the NRS-11 in the first and third months, 77.8%, by the sixth month, and 38.9%, by the twelfth month. Furthermore, at the 6-month mark, the percentage of patients who achieved a 50% or greater reduction in NRS-11 scores was of 100% (18), and by the twelfth month, it was of 66.7%. A successful treatment response correlated with effective diagnostic block and lower frequency of attacks. Differences emerged between patients with NRS-11 scores < 4 and those with scores ≥ 4, with electroshock-like pain more common in those with higher scores and lancinating pain more frequent in those with lower scores. Only 2 patients experienced temporary complications, both of whom recovered quickly.

The pain in ON likely originates from the upper cervical nerves due to convergence between upper cervical segment afferents and the trigeminal complex. 22 Consequently, GON block and RF are the cornerstone of ON treatment. 23 24 25

Studies 5 6 7 8 9 10 on PRF of the DGON with anatomical landmark-guidance have shown significant improvement in pain and quality of life. Choi et al. 7 found PRF more effective than steroid injections for pain reduction in a 6-month double-blinded study. In ON patients, PRF treatments have also been shown to improve sleep, mood, and daily functioning. 14

Only 3 previous studies 12 13 14 have evaluated CRFA's effectiveness in ON. Hoffman et al. 12 reported a 76.3% success rate with DGON RFA using anatomical landmarks over six months. Finiels and Batifol 13 showed a success rate of 89.4% when treating ON with bilateral DGON and C2 DRG CRF under fluoroscopy. 13 In a retrospective observational study of fluoroscopy-guided C2 DRG CRF, Hamer and Purath 14 reported that 35% of the patients experienced complete pain relief, while 70% expreinced at least 80% of pain relief over an average of 22.35 weeks.

In the current study, CRFA targeted the PGON, which is located between the obliquus capitis inferior and semispinalis capitis muscles at C2, a site considered safer and more accessible than that of the DGON. This position enables US guidance, reducing the risks associated with fluoroscopy. 15 16 17 18 Up to 90% of the cases 22 show compression of the GON by surrounding structures, such as the inferior oblique capitis and semispinal capitis muscles or the occipital artery. Successful treatments with US-guided PGON blocks have been documented, although fewer studies have addressed PGON PRF in ON. 15 16 23 24 25 26 Guner and Eyigor 19 reported improvements in depression, migraine symptoms, and sleep disturbances in chronic migraine patients treated with US-guided PGON PRF.

Compared to the PRF and CRFA outcomes in the literature, in the present study, we observed greater effectiveness and longer-lasting pain relief. Here, success was defined as an NRS-11 score < 4, with baseline scores ranging from 8 to 10. By month 12, the scores ranged from 1 to 6. While a 50% of pain reduction compared to baseline was achieved in 100% (18 patients) of the cases in months 1 through 6, this rate was of 66% at 12 months, showing substantial long-term efficacy. However, we could not definitively determine whether PRF or CRF of the PGON is more beneficial under US guidance. Further research is needed to compare the effectiveness of US-guided PRF and CRFA of the PGON.

In the current study, we also observed fewer and less severe complications compared to previous studies, likely due to factors such as US guidance, dexamethasone administration, and precise targeting of the PGON. 11 While complications like hemiplegia and severe neuropathic pain have been reported 13 with C2 DRG CRFA at 70°C, the present study used a CRFA temperature of 60°C to avoid nerve damage while effectively managing pain. In previous studies 25 on trigeminal neuralgia, it was known that temperatures > 65°C destroy the nerve fibers, which, in turn, can lead to severe complications, such as blindness, deafness, ptosis, and permanent facial nerve palsy. As the current was the first study on US-guided PGON CRFA, the temperature was chosen carefully to ensure pain relief and reduce complications. Future research could explore temperature variations to further optimize CRFA outcomes and minimize complications in ON treatments.

## STRENGTHS AND LIMITATIONS

The present study has several notable strengths. It is the first study on the efficacy of US-guided PGON CRFA. The 1-year follow-up and the use of validated instruments such as the HIT-6 enabled an effective evaluation of the long-term outcomes. However, there are also limitations, and the most important limitation one is the retrospective design, with a small sample size and no control group. Additionally, there is the inability to assess sample size or do post-hoc power analysis. In addition, ON can affect various aspects of a patient's quality of life, such as mood, mobility, and sleep, but no data on quality of life or disability were collected in the current study. Psychological factors that may influence the effectiveness of treatments for chronic pain were only assessed using medical records, without detailed examinations. Larger, prospective, controlled studies are needed to better understand the factors that predict the success of CRFA treatment in ON.

In conclusion, the present study showed that US-guided CRFA of the PGON significantly reduces pain in patients with refractory ON up to 1 year after the procedure. The initial attack frequency, electric shock-like pain, and reduced response to diagnostic block were associated with reduced response. These results suggest that CRFA of the PGON is promising. However, before it becomes widely accepted, further scientific studies and randomized controlled trials are needed.
